# Supplementary material for: Unveiling the Bioactive Potential of the Invasive Jellyfish Phyllorhiza punctata Through Integrative Transcriptomic and Proteomic Analyses
Source: Biomolecules. 2025 Aug 4;15(8):1121. doi: 10.3390/biom15081121 (PMC12383608; doi:10.3390/biom15081121)
Supplement: Supplementary file 1 [file biomolecules-15-01121-s001.zip › Suplementary Material/FileS3/PEPFOLD4/XP_065070341_14194/clusters.html]

 PEP-FOLD clusters


Download archive of all models  here   

|  |  |  |
| --- | --- | --- |
| # | file | sOPEP |
| # cluster 1 - Size: 12 |  |  |
|  | XP\_065070341\_14194-model1 | -15.1549 |
|  | XP\_065070341\_14194-model1.1 | -15.1549 |
|  | XP\_065070341\_14194-model1.2 | -15.1549 |
|  | XP\_065070341\_14194-model1.3 | -15.1549 |
|  | XP\_065070341\_14194-model1.4 | -15.0143 |
|  | XP\_065070341\_14194-model1.5 | -14.9846 |
|  | XP\_065070341\_14194-model1.6 | -14.9783 |
|  | XP\_065070341\_14194-model1.7 | -14.9155 |
|  | XP\_065070341\_14194-model1.8 | -14.8534 |
|  | XP\_065070341\_14194-model1.9 | -14.8194 |
|  | XP\_065070341\_14194-model1.10 | -14.5128 |
|  | XP\_065070341\_14194-model1.11 | -14.2094 |
| # cluster 2 - Size: 2 |  |  |
|  | XP\_065070341\_14194-model2 | -14.8289 |
|  | XP\_065070341\_14194-model2.1 | -14.7526 |
| # cluster 3 - Size: 1 |  |  |
|  | XP\_065070341\_14194-model3 | -13.8989 |
| # cluster 4 - Size: 1 |  |  |
|  | XP\_065070341\_14194-model4 | -8.11812 |
| # cluster 5 - Size: 9 |  |  |
|  | XP\_065070341\_14194-model5 | -8.07138 |
|  | XP\_065070341\_14194-model5.1 | -7.99991 |
|  | XP\_065070341\_14194-model5.2 | -7.8117 |
|  | XP\_065070341\_14194-model5.3 | -7.75791 |
|  | XP\_065070341\_14194-model5.4 | -7.55286 |
|  | XP\_065070341\_14194-model5.5 | -7.11681 |
|  | XP\_065070341\_14194-model5.6 | -7.06025 |
|  | XP\_065070341\_14194-model5.7 | -5.77961 |
|  | XP\_065070341\_14194-model5.8 | -5.18405 |
| # cluster 6 - Size: 4 |  |  |
|  | XP\_065070341\_14194-model6 | -7.92002 |
|  | XP\_065070341\_14194-model6.1 | -7.3215 |
|  | XP\_065070341\_14194-model6.2 | -7.17915 |
|  | XP\_065070341\_14194-model6.3 | -5.19221 |
| # cluster 7 - Size: 11 |  |  |
|  | XP\_065070341\_14194-model7 | -7.7693 |
|  | XP\_065070341\_14194-model7.1 | -6.87435 |
|  | XP\_065070341\_14194-model7.2 | -6.78634 |
|  | XP\_065070341\_14194-model7.3 | -6.71278 |
|  | XP\_065070341\_14194-model7.4 | -6.4957 |
|  | XP\_065070341\_14194-model7.5 | -6.21802 |
|  | XP\_065070341\_14194-model7.6 | -6.08406 |
|  | XP\_065070341\_14194-model7.7 | -6.00771 |
|  | XP\_065070341\_14194-model7.8 | -5.7465 |
|  | XP\_065070341\_14194-model7.9 | -5.11538 |
|  | XP\_065070341\_14194-model7.10 | -4.45473 |
| # cluster 8 - Size: 4 |  |  |
|  | XP\_065070341\_14194-model8 | -7.69729 |
|  | XP\_065070341\_14194-model8.1 | -7.42195 |
|  | XP\_065070341\_14194-model8.2 | -7.39927 |
|  | XP\_065070341\_14194-model8.3 | -5.04912 |
| # cluster 9 - Size: 3 |  |  |
|  | XP\_065070341\_14194-model9 | -7.6888 |
|  | XP\_065070341\_14194-model9.1 | -6.69416 |
|  | XP\_065070341\_14194-model9.2 | -4.95636 |
| # cluster 10 - Size: 4 |  |  |
|  | XP\_065070341\_14194-model10 | -7.30325 |
|  | XP\_065070341\_14194-model10.1 | -6.66158 |
|  | XP\_065070341\_14194-model10.2 | -6.58684 |
|  | XP\_065070341\_14194-model10.3 | -6.12318 |
| # cluster 11 - Size: 2 |  |  |
|  | XP\_065070341\_14194-model11 | -7.08555 |
|  | XP\_065070341\_14194-model11.1 | -6.1528 |
| # cluster 12 - Size: 1 |  |  |
|  | XP\_065070341\_14194-model12 | -7.0697 |
| # cluster 13 - Size: 1 |  |  |
|  | XP\_065070341\_14194-model13 | -6.61332 |
| # cluster 14 - Size: 12 |  |  |
|  | XP\_065070341\_14194-model14 | -6.60266 |
|  | XP\_065070341\_14194-model14.1 | -6.5237 |
|  | XP\_065070341\_14194-model14.2 | -6.52152 |
|  | XP\_065070341\_14194-model14.3 | -6.41891 |
|  | XP\_065070341\_14194-model14.4 | -6.24572 |
|  | XP\_065070341\_14194-model14.5 | -5.90225 |
|  | XP\_065070341\_14194-model14.6 | -5.78188 |
|  | XP\_065070341\_14194-model14.7 | -5.60573 |
|  | XP\_065070341\_14194-model14.8 | -5.42063 |
|  | XP\_065070341\_14194-model14.9 | -5.3996 |
|  | XP\_065070341\_14194-model14.10 | -5.365 |
|  | XP\_065070341\_14194-model14.11 | -4.75433 |
| # cluster 15 - Size: 3 |  |  |
|  | XP\_065070341\_14194-model15 | -6.56285 |
|  | XP\_065070341\_14194-model15.1 | -5.83925 |
|  | XP\_065070341\_14194-model15.2 | -5.13291 |
| # cluster 16 - Size: 1 |  |  |
|  | XP\_065070341\_14194-model16 | -6.29699 |
| # cluster 17 - Size: 1 |  |  |
|  | XP\_065070341\_14194-model17 | -6.02519 |
| # cluster 18 - Size: 2 |  |  |
|  | XP\_065070341\_14194-model18 | -5.8636 |
|  | XP\_065070341\_14194-model18.1 | -4.59944 |
| # cluster 19 - Size: 2 |  |  |
|  | XP\_065070341\_14194-model19 | -5.30708 |
|  | XP\_065070341\_14194-model19.1 | -4.67777 |
| # cluster 20 - Size: 1 |  |  |
|  | XP\_065070341\_14194-model20 | -5.20975 |
| # cluster 21 - Size: 3 |  |  |
|  | XP\_065070341\_14194-model21 | -4.55532 |
|  | XP\_065070341\_14194-model21.1 | -4.13029 |
|  | XP\_065070341\_14194-model21.2 | -4.0739 |
| # cluster 22 - Size: 1 |  |  |
|  | XP\_065070341\_14194-model22 | -4.34447 |
| # cluster 23 - Size: 2 |  |  |
|  | XP\_065070341\_14194-model23 | -4.31568 |
|  | XP\_065070341\_14194-model23.1 | -4.10401 |
| # cluster 24 - Size: 1 |  |  |
|  | XP\_065070341\_14194-model24 | -4.28178 |
| # cluster 25 - Size: 2 |  |  |
|  | XP\_065070341\_14194-model25 | -4.21609 |
|  | XP\_065070341\_14194-model25.1 | -4.14852 |
| # cluster 26 - Size: 1 |  |  |
|  | XP\_065070341\_14194-model26 | -4.16708 |
| # cluster 27 - Size: 2 |  |  |
|  | XP\_065070341\_14194-model27 | -4.1197 |
|  | XP\_065070341\_14194-model27.1 | -4.08755 |
| # cluster 28 - Size: 1 |  |  |
|  | XP\_065070341\_14194-model28 | -4.10393 |
| # cluster 29 - Size: 1 |  |  |
|  | XP\_065070341\_14194-model29 | -4.09281 |
| # cluster 30 - Size: 2 |  |  |
|  | XP\_065070341\_14194-model30 | -4.08793 |
|  | XP\_065070341\_14194-model30.1 | -4.01154 |
| # cluster 31 - Size: 1 |  |  |
|  | XP\_065070341\_14194-model31 | -4.07053 |
| # cluster 32 - Size: 1 |  |  |
|  | XP\_065070341\_14194-model32 | -4.05542 |
| # cluster 33 - Size: 1 |  |  |
|  | XP\_065070341\_14194-model33 | -4.04566 |
| # cluster 34 - Size: 1 |  |  |
|  | XP\_065070341\_14194-model34 | -4.03657 |
| # cluster 35 - Size: 1 |  |  |
|  | XP\_065070341\_14194-model35 | -4.01021 |
| # cluster 36 - Size: 1 |  |  |
|  | XP\_065070341\_14194-model36 | -3.98247 |
| # cluster 37 - Size: 1 |  |  |
|  | XP\_065070341\_14194-model37 | -3.94282 |
|  |  |  |
